# FDG uptake in upper abdominal lymph node as a distinctive pattern in sarcoidosis

**DOI:** 10.1007/s11604-025-01935-x

**Published:** 2026-02-05

**Authors:** Ryogo Minamimoto, Yumi Abe, Ryota Morimoto, Rintato Ito, Naotoshi Fujita, Toyoaki Murohara, Katsuhiko Kato, Shinji Naganawa

**Affiliations:** 1 Department of Integrated Image Information Analysis, Nagoya University Graduate School of Medicine, 65, Tsurumaicho, Shouwa-ku, Nagoya, Aichi 466-8550 Japan; 2 Department of Radiology, Nagoya University Graduate School of Medicine, Nagoya, Aichi Japan; 3 Department of Cardiology, Nagoya University Graduate School of Medicine, Nagoya, Japan; 4 Department of Innovative Biomedical Visualization (iBMV), Nagoya University Graduate School of Medicine, Nagoya, Aichi Japan; 5 Department of Radiological Technology, Nagoya University Hospital, Nagoya, Aichi Japan

**Keywords:** Sarcoidosis, FDG, PET/CT, Abdominal, Lymph node

## Abstract

**Objectives:**

To evaluate the distribution patterns of sarcoidosis involvement on FDG-PET/CT in patients with known or suspected cardiac sarcoidosis (CS), with a particular focus on upper abdominal lymph nodes (LN) (periportal LN [PLN], anterior pancreaticoduodenal LN [APDLN], and posterior pancreaticoduodenal LN [PPDLN]) and the association of them with other lesions and myocardium.

**Methods:**

We identified 861 FDG-PET/CT scans performed between July 2016 and August 2024 in patients with known or suspected CS, and included 178 cases for analysis of FDG uptake patterns suggestive of sarcoid involvement. FDG-positive LNs or regions were classified as sarcoidosis-related based on treatment response, characteristic uptake patterns, or histological confirmation. The occurrence ratio of FDG-positive lymph nodes or regions was also assessed in relation to myocardial FDG uptake patterns.

**Results:**

FDG uptake was observed most frequently in hilar and mediastinal LNs (79% and 76%, respectively). Upper abdominal LN uptake was observed in 49.4% of patients, most commonly in the PLN (31.5%), APDLN (38.2%), and PPDLN (37.1%). Heatmap analyses revealed strong co-occurrence between thoracic and upper abdominal LNs, suggesting a lymphatic dissemination pattern. Peripheral LNs such as axillary, subclavian, and inguinal/pelvic stations demonstrated low uptake and minimal co-occurrence.

**Conclusions:**

FDG-PET/CT provides valuable insight into the structured lymphatic dissemination of sarcoidosis. Frequent FDG uptake in upper abdominal lymph nodes, particularly when accompanied by thoracic involvement, represents a characteristic finding in sarcoidosis. Recognition of this pattern can improve diagnostic accuracy and help differentiate sarcoidosis from other systemic diseases.

**Secondary abstract:**

This study assessed lymph node involvement on FDG-PET/CT in patients with suspected or known cardiac sarcoidosis, revealing distinct dissemination patterns into the upper abdomen. These findings enhance understanding of disease pathophysiology and may improve diagnostic evaluation.

## Introduction

Sarcoidosis is a multisystem granulomatous disease that has nonspecific clinical manifestations that commonly affect the pulmonary system in at least 90% of sarcoidosis patients. In 10–30% of cases there is involvement of other organs such as the eyes, skin, liver, spleen, and lymph nodes (LNs). The clinical presentation varies depending on the involved organ. Various factors, including infection, genetic predisposition, and environmental factors, are involved in the pathology of sarcoidosis [1]. Sarcoidosis presents with a wide range of clinical assignations that range from asymptomatic to fatal. Although its etiology remains unknown, some studies have reported that processing of an unidentified antigen by activated macrophages instigates an immune response that is regulated by T-cells and macrophages. These activated cells discharge various mediators, including cytokines, chemokines, and reactive oxygen species that may be involved in progression of the disease [1]. Among diagnostic imaging modalities, ^18^F-fluorodeoxy glucose (FDG) positron emission tomography (PET)/computed tomography (CT) is considered the most effective for detecting tissue-specific inflammatory activity and identifying sites for diagnostic biopsies [1, 2]. FDG-PET is widely accepted as the most suitable modality for identifying the active area of inflammation in patients diagnosed with or clinically suspected to have cardiac sarcoidosis (CS) [3] as it enables confirmation of FDG uptake throughout the body as a characteristic finding suggesting the involvement of sarcoidosis. In our experience of FDG-PET and FDG-PET/CT investigations, we commonly observe focal FDG uptake in the periportal LNs (PLNs), anterior pancreaticoduodenal LNs (APDLNs), and posterior pancreaticoduodenal LNs (PPDLNs), and these areas sometimes connect. We have observed this pattern more frequently than FDG uptake in celiac LNs. Warchauer and Lee reported several imaging patterns of abdominal sarcoidosis on CT and/or ultrasound (US) [4] but did not specify the origin of abdominal sarcoidosis, and no studies have reported the findings on FDG-PET/CT. The aim of the present study was to investigate the frequency of each pattern of LN involvement on FDG-PET/CT in patients with sarcoidosis, and evaluate their typical features.

## Materials and methods

### Study design

This study was performed in line with the principles of the Declaration of Helsinki. This study was approved by the Ethics Review Committee, Nagoya University Graduate School of Medicine (approval No. 2024-0259), which waived the requirement for informed consent. We conducted a retrospective review of consecutive patients who underwent PET/CT for known or suspected CS between July 2016 and August 2024. A total of 861 FDG-PET/CT studies were identified during this period.

### Subjects

The diagnosis or suspicion of CS prior to FDG-PET/CT was based on the following criteria established by the Japanese Ministry of Health [5]: high-grade atrioventricular block or life-threatening ventricular arrhythmia; basal thinning of the ventricular septum or other abnormal ventricular wall morphologies (e.g., ventricular aneurysm, thinning of the mid or upper septum, regional wall thickening); and left ventricular dysfunction with an ejection fraction below 50%. Histological diagnosis, cardiac MRI with late gadolinium enhancement, and pathological confirmation via tissue biopsy were also investigated when available. The present study cohort included some cases that were also analyzed in a different study that evaluated the efficacy of MTX or repeat PSL for active CS [6]. All FDG-PET/CT images analyzed in this study were obtained before initiation of systemic treatment such as corticosteroids or immunosuppressants, and thus reflect the untreated disease status.

### FDG PET/CT acquisition

To minimize physiological myocardial FDG uptake, patients were instructed to follow a carbohydrate-restricted, high-protein diet for one week, avoid vigorous exercise for 24 h prior to the scan, and fast for at least 18 h before the examination. Blood glucose and free fatty acid levels were measured immediately before FDG administration to confirm adherence to the fasting protocol. FDG was administered intravenously with a dose of 3.7 MBq/kg for patients weighing < 60 kg, and 4.07 MBq/kg for those weighing ≥ 60 kg.

All FDG PET/CT scans were performed using either a SIEMENS Biograph 16 or SIEMENS Biograph Horizon scanner (Siemens Healthineers, Erlangen, Germany). Image acquisition began 60 min after FDG injection, covering the area from the base of the skull to the proximal femora, followed by a dedicated cardiac PET scan. For the Biograph 16, scans were acquired in step-and-shoot mode with an acquisition time of 1.7–2.0 min per bed position. For the Biograph Horizon, scans were acquired using either SS mode or continuous bed motion mode, with a table speed of 0.8–1.5 mm/sec, including dynamic acquisitions. Breath-holding and respiratory gating were not employed. PET images were reconstructed using a Gaussian filter (5.0 mm full-width at half maximum) and the ordered-subset expectation maximization algorithm, incorporating a CT-based attenuation correction map. Images were reconstructed at a matrix size of 256 × 256 pixels (Biograph 16) or 180 × 180 pixels (Biograph Horizon). We confirmed that the recovery coefficients were nearly equivalent among the PET/CT scanners based on a NEMA phantom study.

### Determination of FDG-positive lymph nodes or regions as sarcoidosis involvement

Compared to baseline FDG-PET/CT, LNs that exhibited markedly decreased or resolved FDG uptake on PET/CT following therapy for CS, compared to baseline FDG-PET/CT were interpreted as sites of sarcoid involvement, and the baseline scan was used to evaluate the distribution of sarcoidosis in this study, If FDG uptake in swollen bilateral hilar LNs and/or symmetrical FDG uptake in swollen mediastinal LNs were present, which are considered characteristic findings of sarcoidosis, other LNs that showed high FDG uptake were also presumed to be involved. When histological confirmation of sarcoidosis was obtained from percutaneous or surgical biopsy specimens, other LNs with intense FDG uptake were similarly classified as sarcoidosis-related.

### Image interpretation

All PET/CT images were reviewed using a SAI viewer (FUJIFILM Medical Co., Ltd., Tokyo, Japan), which is routinely employed in daily clinical practice, by two board-certified nuclear medicine physicians (RM and YA) in consensus. Focal FDG uptake greater than background uptake was defined as a positive region. Particular attention was given to FDG uptake in the PLN, APDLN, PPDLN and celiac LN on fused PET/CT images. For each LN, the maximum standardized uptake value (SUV_max_) was recorded by placing a spherical volume of interest around the LNs using Syngo.via workstation (Siemens Healthineers). The largest axial diameter of each LN was measured on the CT component of the PET/CT scan by a board-certified radiologist (RM) and, when available, re-evaluated on diagnostic chest CT images acquired within two months of the PET/CT, using the SAI viewer. Myocardial FDG uptake patterns were evaluated based on the findings described in the diagnostic reports and classified into three categories: negative FDG uptake, focal FDG uptake (including focal on diffuse patterns), and diffuse uptake.

### Data analysis and statistical analysis

In this study, upper abdominal LNs refer specifically to the following three regions: PLN, APDLN, and PPDLN, and do not include celiac and para-aortic lymph nodes. Co-occurrence of FDG-positive LNs across anatomical regions was evaluated by constructing a co-occurrence matrix, where each cell represented the number of cases with simultaneous FDG uptake in the corresponding nodal regions. A heatmap was generated using Excel, with color intensity indicating the frequency of co-occurrence.

Independent two-sample t-tests were conducted between each FDG-avid LN in the abdomen. The association between upper abdominal LN uptake and hilar or mediastinal LN uptake was assessed across cardiac uptake patterns using chi-square tests. A p-value of less than 0.05 was considered statistically significant. All statistical analyses were performed using Stata 15 (Stata Corp LLC).

## Results

For the 861 FDG-PET/CT studies performed in patients with known or suspected CS, patient records were reviewed and the clinical background and prognosis were assessed. Thirty-five cases were ultimately diagnosed as neither CS nor systemic sarcoidosis. Among the remaining studies, 517 examinations were follow-up FDG-PET/CT scans (third or subsequent examinations) in patients with suspected or known or suspected CS, and 131 were second-time FDG-PET/CT scans used to assess therapeutic response to CS compared to initial FDG-PET/CT scans. Finally, this study analyzed a total of 178 patients (mean age ± SD: 62.4 ± 12.3 years; range: 26–88 years; female: 129; male: 49). Of these, 47 patients underwent a single FDG-PET/CT scan, and 131 patients had an initial FDG-PET/CT followed by a second examination. Any FDG uptake in myocardium was confirmed in 79.1% (139/178) of patients. Among the 39 cases without myocardial FDG uptake, sarcoidosis was confirmed based on the CS diagnostic criteria (n = 11), biopsy results (lung: 17, eye: 11, skin: 7, liver: 1, kidney: 1, muscle: 1), or the presence of bilateral hilar lymphadenopathy indicative of sarcoidosis (n = 7). Based on the guideline-based diagnostic criteria [5], 155 of the 178 patients (87.1%) were diagnosed with CS. The remaining 23 patients underwent FDG-PET to identify potential myocardial inflammatory sites of sarcoidosis because they had histopathologically confirmed extracardiac sarcoidosis and/or clinical features consistent with sarcoidosis in the lungs, hilar or mediastinal lymph nodes, or eyes, but did not meet the full diagnostic criteria for cardiac involvement. Patient characteristics and study details are shown in Table 1.

**Table 1:** Baseline patient characteristics and study details

Index	Value
Age (Mean ± SD)	62.4 ± 12.3
Age range	26–88
Sex (Female: Male)	129 (72.5%):49 (27.5%)
Incidence according to diagnostic criteria of cardiac sarcoidosis	High-grade atrioventricular block or life-threatening ventricular arrhythmia: 87 (48.9%) Basal thinning of the ventricular septum or other abnormal ventricular wall morphologies: 74 (41.6%) Left ventricular dysfunction with an ejection fraction below 50%: 79 (44.4%) Cardiac MRI with late gadolinium enhancement: 52 (29.2%) Histological diagnosis: lung or mediastinum: 110 (61.8%), skin: 26 (14.6%), eye: 35 (19.7%), others: 21(11.8%) Myocardial biopsy: 8 (4.5%)
FDG-PET/CT scan	Initial FDG-PET/CT study only: 47 (26.4%) Initial + second follow up scan: 131 (73.6%)
Confirmation of sarcoidosis in cases with negative myocardial FDG uptake (n = 39)	Cardiac sarcoidosis diagnostic criteria: 11 Biopsy results: 21 (lung or mediastinum: 17, eye: 11, skin: 7, liver: 1, kidney: 1, muscle: 1) Bilateral hilar lymphadenopathy: 7

### FDG-PET/CT findings

**Fig. 1:**
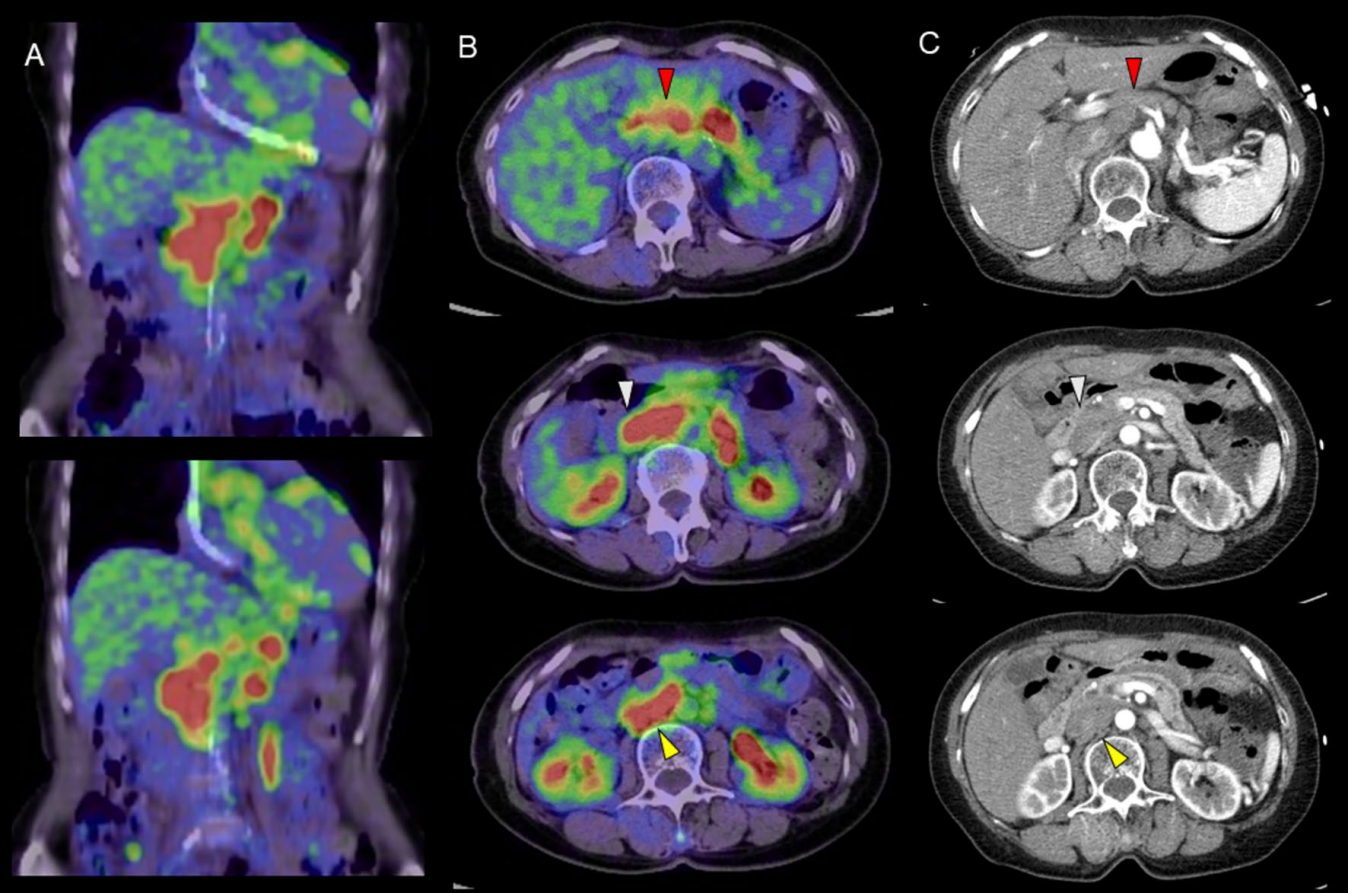
**A**Coronal images of fused PET/CT image, **B** axial images of fused PET/CT, **C** axial images of contrast CT. Intense FDG uptake was observed in multiple swollen lymph nodes: the periportal lymph node (upper row, red arrowheads), anterior pancreaticoduodenal lymph node (middle row, white arrowheads), and posterior pancreaticoduodenal lymph node (lower row, yellow arrowheads), along with uptake in the celiac lymph node

**Fig. 2:**
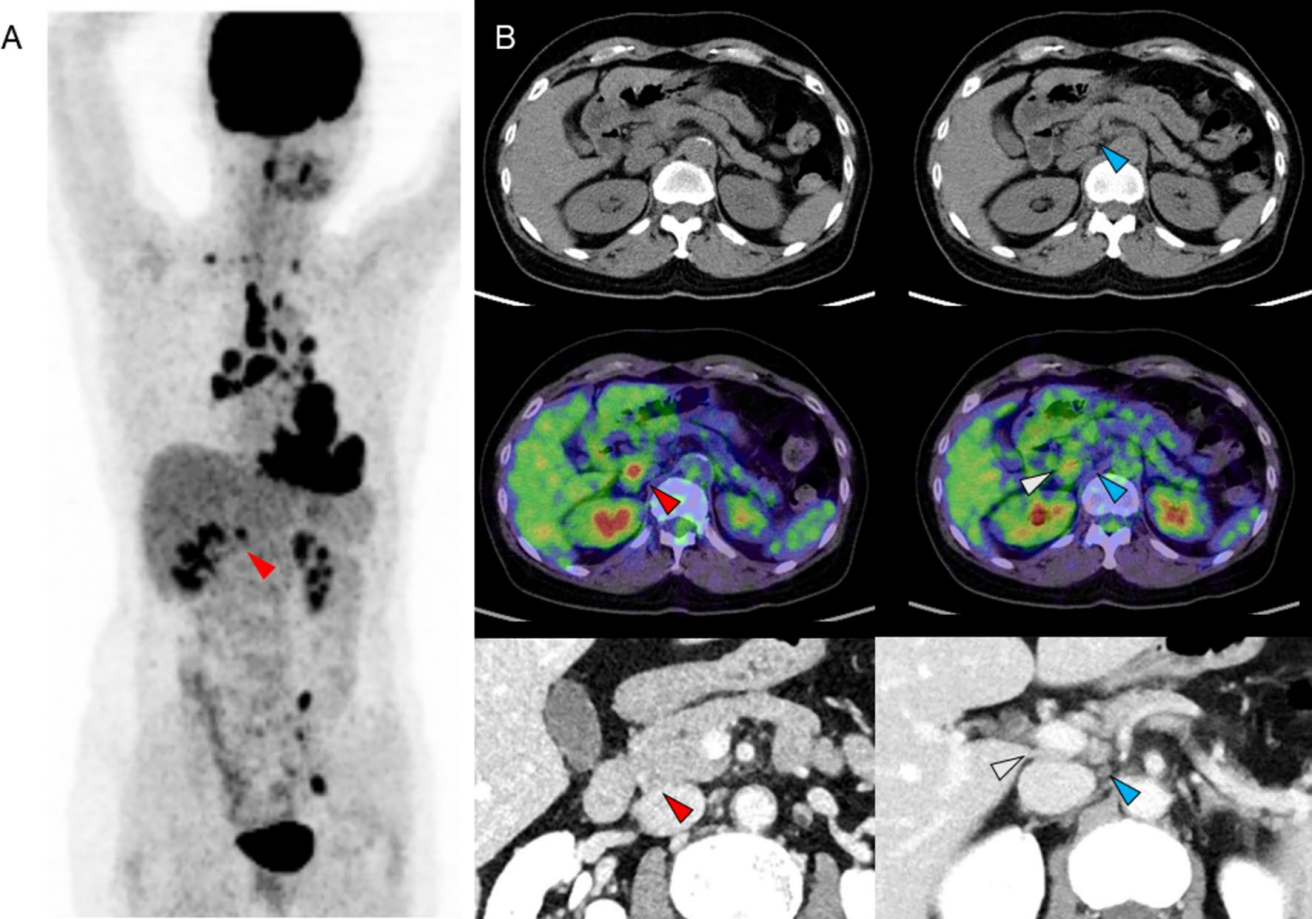
**A** Maximum intensity projection (MIP) image of FDG-PET, **B** upper row: CT portion of PET/CT, middle row: fused PET/CT image, lower row: contrast CT image. Focal FDG uptake corresponding to a pancreaticoduodenal lymph node is seen (red arrowhead), identifiable as a small nodule on the CT component of PET/CT, and slightly more conspicuous on contrast-enhanced CT. In contrast, a small periportal lymph node (blue arrowhead) is visualized on contrast-enhanced CT, but shows no FDG uptake. The MIP image also shows intense FDG uptake in the hilar and mediastinal lymph nodes, as well as in the myocardium, suggestive of sarcoidosis involvement

**Fig. 3:**
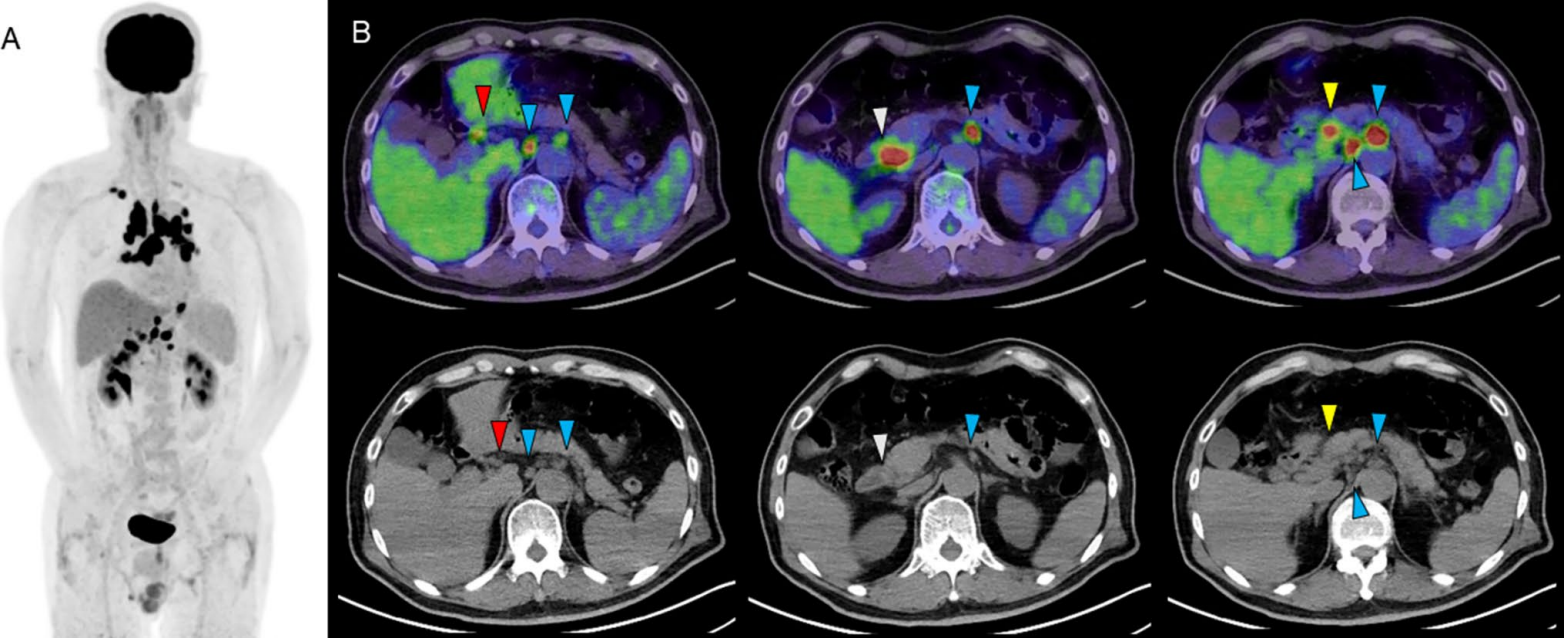
**A** Maximum intensity projection (MIP) image of FDG-PET, **B** upper row: fused PET/CT image, lower row: CT portion of PET/CT. The MIP image demonstrates intense FDG uptake in the hilar and mediastinal lymph nodes, as well as in multiple lymph nodes (LNs) in the upper abdominal region. In contrast, no abnormal FDG uptake was observed in the myocardium. Focal FDG uptake was seen in periportal LN (red arrow heads), anterior pancreaticoduodenal lymph node (yellow arrowheads), posterior pancreaticoduodenal LN (white arrowheads), and celiac LNs (blue arrowheads) was seen. Although these lymph nodes are small and exhibit benign morphological features on CT, intense FDG uptake is commonly observed in patients with sarcoidosis

Table 2 summarizes the FDG uptake value in regional LN and organs suggesting involvement of sarcoidosis. The hilar and mediastinal LN FDG uptake was detected in 78.7% (140/178) and 75.8% (135/178) of cases, with mean SUV_max_ of 6.7 ± 2.9 (range: 2.2–19.4) and 7.7 ± 3.6 (range: 2.8–28.9), respectively. Upper abdominal LN uptake was present in 49.4% (88/178), with a mean SUV_max_ of 7.0 ± 3.7 (range: 2.1–22.1). FDG uptake in upper abdominal regions included the PLN (31.5%, 56/178), APDLN (38.2%, 68/178), and PPDLN (37.1%, 66/178) (no significant difference). Among the upper abdominal regions, three positive areas were found in 44.3% of patients, two positive areas in 26.1% (PLN-APDLN: 6.8%, PLN-PPDLN: 5.7%, APDLN-PPDLN: 13.6%), and one positive area in 29.5% (PLN: 6.8%, APDLN: 12.5%, PPDLN: 10.2%) were confirmed. The corresponding mean LN size was 16 ± 6 mm (range: 6–39 mm) in the liver area, 16 ± 7 mm (range: 4–44 mm) in the duodenal area, and 18 ± 7 mm (range: 6–36 mm) in the pancreatic area. These lymph nodes tended to exhibit a flattened shape and were difficult to identify on CT without the guidance of FDG uptake. Representative FDG-PET/CT findings are illustrated in Figs. 1, 2 and 3.

**Table 2:** FDG uptake value in regional LN and organs suggesting involvement of sarcoidosis

	Head and neck	Subclavian	Axillary	Lung	Hilar	Media	Celiac	Para aorta	Upper abdominal
Mean SUV_max_ (± SD)	4.1	6.2 ± 2.1	3.9 ± 1.0	7.3 ± 6.3	6.7 ± 2.9	7.7 ± 3.6	6.3 ± 3.2	7.5 ± 3.5	7.0 ± 3.7
Range	3.3–4.9	2.6–5.2	2.5–18.3	1.9–23.6	2.2–19.4	2.8–28.9	2.5–18.3	3.3–20.9	2.1–22.1
Number of positive cases	2	20	5	18	140	135	51	42	88
Positive ratio (%)	1.1	11.2	2.8	10.1	78.7	75.8	28.7	23.6	49.4

	Upper abdominal			Liver	Spleen	Kidney	Inguinal /pelvic	Bone
	PLN	APDLN	PPDLN					
Mean SUV_max_ (± SD)	7.0 ± 3.3	7.1 ± 4.0	6.9 ± 3.8	7.2 ± 2.3	6.4 ± 3.3	9.1	8.3 ± 5.6	11.9 ± 6.8
Range	2.1–18.5	2.7–19.6	2.5–22.1	4.2–12.7	2.3–18.2	9.1	3.0–19.5	7.4–21.9
Number of positive cases	56	68	66	13	36	1	11	4
Positive ratio (%)	31.5	38.2	37.1	7.3	20.2	0.6	6.2	2.2

*PLN* periportal lymph node, *APDLN* anterior pancreaticoduodenal lymph node, *PPDLN* posterior pancreaticoduodenal lymph node. Upper abdominal: combined PLN, APDLN and PPDLN

Additional nodal involvement was observed in the paraaortic region (23.6%, 42/178), subclavian region (11.2%, 20/178), inguinal/pelvic region (6.2%, 11/178) and axillary region (2.8%, 5/178), and. FDG uptake in solid organs was noted in the spleen (20.2%, 36/178), lungs (10.1%, 18/178), liver (7.3%, 13/178), bone (2.2%, 4/178), head and neck region (1.1%, 2/178), and kidneys (0.6%, 1/178).

**Fig. 4:**
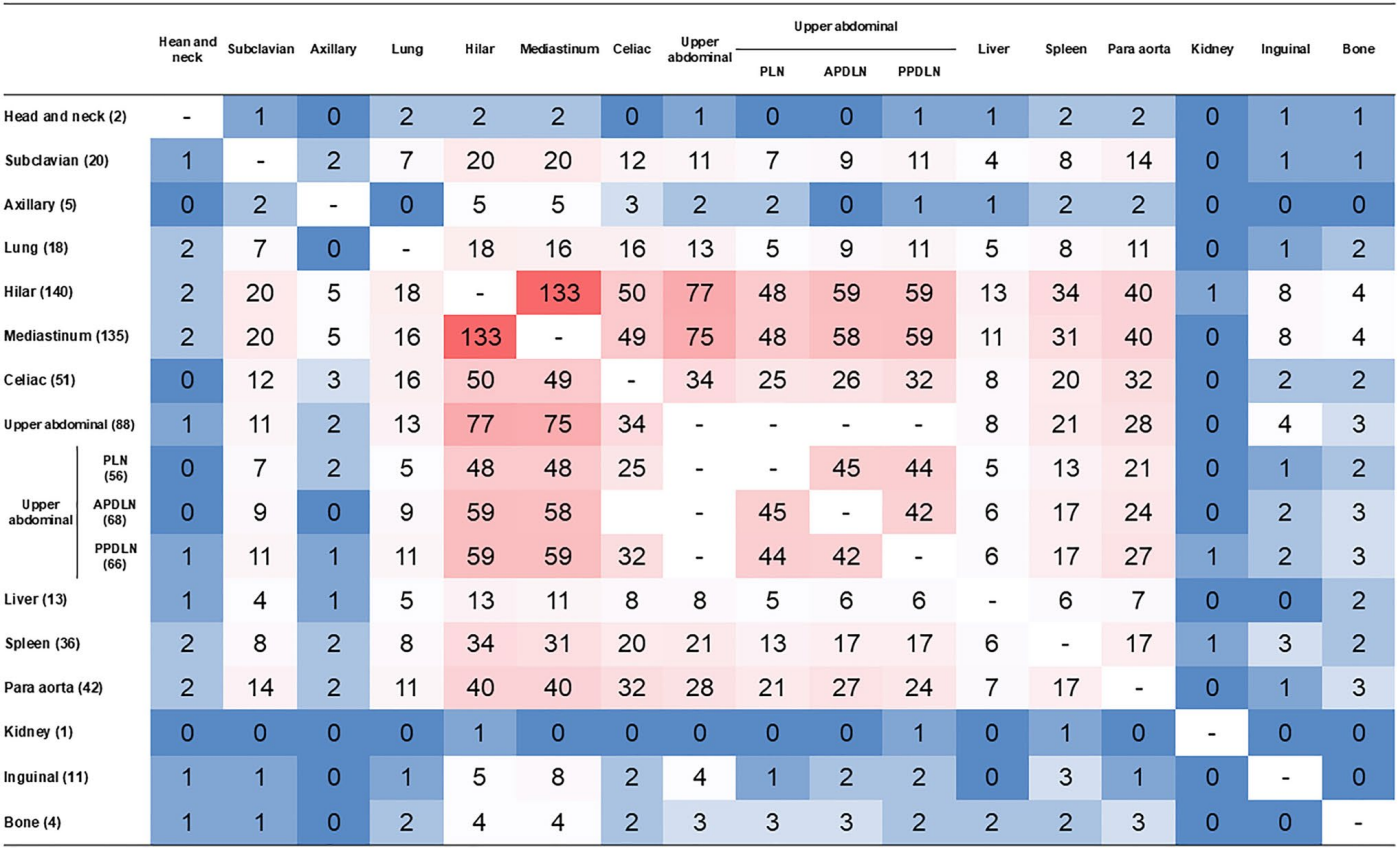
Heatmap illustrating the frequency of co-occurrence of FDG-positive regions in sarcoidosis patients. Each cell represents the number of patients with simultaneous FDG uptake in the row and column regions. The color scale reflects the frequency of co-occurrence: red tones indicate higher frequencies, while blue tones indicate lower frequencies. White represents minimal or no co-occurrence. The numbers within each cell correspond to the actual case count

The heatmap demonstrated strong co-occurrence patterns between hilar and mediastinal LNs, as well as between upper abdominal LNs and both the hilar and mediastinal regions (Fig. 4).

The co-occurrence rate with hilar and mediastinal regions was higher in upper abdominal LNs than in celiac LNs. Co-positivity was notable between paraaortic and upper abdominal, and celiac LNs. In contrast, co-occurrence frequencies were lower for subclavian, axillary, and inguinal/pelvic regions. These patterns suggest characteristic nodal dissemination pathways related to lymphatic drainage in sarcoidosis. Table 3 presents a comparison of patient characteristics and imaging findings between cases with and without abdominal lymph node FDG uptake.

**Table 3:** Comparison of patient characteristics and imaging findings between cases with and without upper abdominal lymph node FDG uptake

	FDG-positive upper abdominal LN (n = 88)		FDG-negative upper abdominal LN (n = 90)	
Age (Mean ± SD)	63.5 ± 10.8		61.5 ± 12.4	
Age range	31–81		26–88	
Sex (Female: Male)	68 (77.3%):20 (22.7%)		61 (67.8%):29 (32.2%)	

FDG uptake sites	Number	Positive ratio (%)	Number	Positive ratio (%)
Head and neck	1	1.1	1	1.1
Subclavian LN	11	12.5	9	10.0
Axillary LN	2	2.3	3	3.3
Lung	13	14.8	5	5.6
Hilar LN	77	87.5	63	70.0
Mediastinum LN	75	85.2	60	66.7
Celiac LN	34	38.6	17	18.9
Para aorta LN	28	31.8	14	15.6
Liver	8	9.1	5	5.6
Spleen	21	23.9	15	16.7
Kidney	0	0.0	1	1.1
Inguinal/pelvic LN	4	4.5	7	7.8
Bone	3	3.4	1	1.1

Myocardial FDG uptake pattern	Number	Positive ratio (%)	Number	Positive ratio (%)
Negative	14	15.9	25	27.8
Focal	43	48.9	34	37.8
Diffuse	31	35.2	31	34.4

*LN* lymph node, Upper abdominal: combined PLN, APDLN and PPDLN

The incidence of FDG uptake sites was more frequent in cases with FDG-positive abdominal lymph nodes.

### FDG uptake in lesions according to myocardial uptake pattern

Table 4 demonstrate the FDG uptake and occurrence ratio of lesions according to myocardial uptake patterns. FDG uptake in the myocardium was classified as focal in 77 cases, diffuse in 62 cases, and negative in 39 cases. Positive FDG uptake was observed more frequently across most regions in patients who had focal myocardial uptake. However, there was no significant difference in FDG uptake values (SUV_max_) among myocardial uptake patterns across any anatomical region. Occurrence rates were consistently the highest for hilar and mediastinal LNs (> 70–80%) with intense FDG uptake (mean SUV_max_: 6.2–8.7) across all uptake patterns. Upper abdominal LNs showed a moderate frequency of involvement (36–56%), also with high FDG avidity (mean SUV_max_: 6.3–7.7). The focal myocardial uptake pattern was associated with more widespread LN involvement, exhibiting relatively high occurrence rates and FDG uptake in multiple regions. However, there were no definitive differences compared to the negative and diffuse patterns. Overall, no strict correlation was observed between the frequency of uptake and its values. No significant differences were observed in the frequency of upper abdominal LN positivity (*p* = 0.08–0.17), hilar LN uptake (*p* = 0.37), or mediastinal LN uptake (*p* = 0.22) among the different cardiac uptake patterns. There were no statistically significant differences in the co-occurrence rates of positive upper abdominal LN FDG uptake and hilar or mediastinal LN uptake among the cardiac FDG uptake patterns (*p* = 0.259). These findings might suggest that upper abdominal and hilar/mediastinal LN involvement occurs independently of the cardiac FDG uptake pattern. Among 178 cases, 13 cases (7.3%) had no extra-cardiac FDG uptake. Focal myocardial FDG uptake patterns was more common in the FDG positive abdominal LN group (48.9% vs. 37.8%), whereas negative uptake was more frequent in the FDG negative abdominal LN group (27.8% vs. 15.9%) (Table 3).

**Table 4:** FDG uptake and occurrence ratio of lesions according to myocardial uptake patterns

Uptake pattern		Head and neck	Subclavian	Axillary	Lung	Hilar	Media	Celiac	Para aorta	Upper abdominal
Negative (39)	Mean SUV_max_ (± SD)	4.9	5.5 ± 1.3	–	4.6 ± 0.7	7.8 ± 3.9	8.7 ± 4.3	6.3 ± 4.3	7.5 ± 4.4	7.0 ± 3.0
Focal (77)		–	6.7 ± 2.9	4.0 ± 1.3	6.3 ± 5.2	6.7 ± 2.8	7.8 ± 3.9	6.6 ± 3.4	8.2 ± 3.8	7.7 ± 4.6
Diffuse (62)		3.3	5.8 ± 1.2	3.7 ± 0.5	14.5 ± 9.8	6.2 ± 2.2	7.1 ± 2.5	6.0 ± 2.0	6.7 ± 2.6	6.3 ± 3.0
Negative (39)	Occurrence ratio (%)	1.6	12.8	0.0	10.3	71.8	64.1	23.1	17.9	35.9
Focal (77)		0	13.0	3.9	14.3	79.2	80.5	32.5	24.7	55.8
Diffuse (62)		1.6	8.1	3.2	4.8	82.3	77.4	24.2	25.8	50.0

Uptake pattern	Upper abdominal			Liver	Spleen	Kidney	Inguinal/pelvic	Bone
	PLN	APDLN	PPDLN					
Negative (39)	7.7 ± 4.2	6.5 ± 2.4	6.8 ± 2.6	7.2 ± 0.3	7.1 ± 5.0	–	9.5 ± 8.8	–
Focal (77)	7.3 ± 3.8	7.4 ± 4.6	7.6 ± 4.7	7.2 ± 2.6	7.0 ± 2.3	9.1	8.6 ± 5.9	8.6 ± 1.5
Diffuse (62)	6.0 ± 3.3	6.2 ± 2.9	6.4 ± 3.3	7.6	5.5 ± 3.0	–	6.8 ± 1.9	21.9
Negative (39)	23.1	28.2	25.6	5.1	20.5	0.0	7.7	0.0
Focal (77)	36.4	45.5	45.5	13.0	18.2	1.3	6.5	3.9
Diffuse (62)	29.0	33.9	32.3	1.6	21.0	0.0	4.8	1.6

*PLN* portal lymph node, *APDLN* anterior pancreaticoduodenal lymph node, *PPDLN* posterior pancreaticoduodenal lymph node. Upper abdominal: combined PLN, APDLN and PPDLN. Numbers in parentheses indicate the number of cases in each category

## Discussion

This study comprehensively evaluated LN involvement patterns using FDG-PET/CT in patients with known or suspected CS. We identified characteristic dissemination patterns that reflect the underlying lymphatic spread of sarcoidosis into the upper abdominal region. These findings enhance our understanding of the disease pathophysiology and may have important implications for diagnostic assessment. Sarcoidosis is a multisystem granulomatous disease with heterogeneous clinical presentations and variable organ involvement. FDG-PET/CT has proven highly effective in detecting extracardiac inflammatory activity, which is present in up to 97% of patients with CS [7], and in guiding the selection of biopsy sites [8, 9]. FDG-PET/CT has emerged as a critical modality for CS evaluation, providing high sensitivity for the detection of active inflammation in both cardiac and extracardiac sites [10].

In our study, hilar and mediastinal LNs were involved in 79% and 76% of cases, respectively, consistent with prior reports [11]. Simonen et al. reported increased FDG uptake in mediastinal LNs in 63% of CS patients, particularly in the right upper and lower paratracheal, subcarinal, subaortic, and para-aortic regions. In addition, 26% of their cohort showed FDG-avid lesions outside the mediastinum, reinforcing the role of mediastinal LNs as accessible biopsy targets [12]. Teirstein et al. identified 139 cases of extracardiac uptake in their evaluation of 188 FDG-PET scans in 137 sarcoidosis patients. Mediastinal LNs (n = 54), extrathoracic LNs (n = 30), and lung parenchyma (n = 24) were the most frequently involved regions, followed by the spleen, liver, muscle, lacrimal/parotid glands, subcutaneous tissue, and bone [11]. Their findings highlighted the mediastinum as the most common site of extracardiac disease, possibly explained by lymphatic drainage of the left heart to the right upper paratracheal LNs [13].

Beyond thoracic involvement, FDG uptake in upper abdominal LN was identified in over 50% of our cohort. FDG-positive LNs were detected most frequently in the PLN, PPDLN, APDLN and celiac LN regions. Our heatmap analysis further demonstrated frequent co-positivity among anatomically adjacent LNs within established lymphatic territories. For example, the highest co-occurrence was observed between hilar and mediastinal LNs (133 cases), followed by upper abdominal–hilar (88 cases) and upper abdominal–mediastinal (85 cases) associations. These findings support the hypothesis that sarcoidosis spreads through lymphatic channels. In contrast, peripheral LNs such as the axillary, subclavian, inguinal/pelvic, and bone marrow regions showed minimal co-occurrence with central LNs.

Although intra-abdominal sarcoidosis is relatively uncommon, it can occur even in the absence of thoracic or pulmonary disease. It is often asymptomatic but may lead to serious complications if left undiagnosed [14]. Abdominal lymphadenopathy is observed in approximately 30% of sarcoidosis patients, typically affecting the hepatic hilum, celiac axis, para-aortic area, mesentery, and iliac vessels. CT imaging shows hypodense LNs, generally 1–2 cm in diameter. LNs exceeding 2 cm are seen in up to 10% of cases and may raise concern for malignancy, particularly lymphoma [15–17].

However, due to their small size, benign appearance, and complex anatomical location, upper abdominal LNs have received limited attention, despite prior reports identifying them as a typical site of sarcoid involvement on CT imaging. Our findings provide additional evidence that FDG uptake in upper abdominal LNs is a specific and diagnostically useful feature of sarcoidosis.

Britt et al. have reported that sarcoid LNs were smaller and less confluent in sarcoidosis than in non-Hodgkin lymphoma (NHL). The average nodal size was significantly smaller in sarcoidosis (2.6 ± 1.7 cm) than in NHL (8.0 ± 5.5 cm), in which LNs were more frequently located in the retrocrural space [17]. In another study, assessment based on mediastinal LN FDG uptake showed no significant differences between sarcoidosis and sarcoid reaction, or between sarcoid reaction and malignant lymphoma [18]. Therefore, FDG uptake in upper abdominal LNs may serve as a key finding to support a more confident diagnosis of sarcoidosis. Involvement of PLNs, PPDLNs, APDLNs, and celiac LNs occurs because of their roles in the lymphatic drainage of the pancreas and liver. Celiac LNs, located at the root of the celiac artery, receive lymph drainage from the stomach and spleen. Duodenal lymphatics drain into pancreaticoduodenal and pyloric LNs (anterior group), or via the head of the pancreas to superior mesenteric LNs (posterior group), with eventual convergence toward celiac LNs [19, 20].

Several studies have proposed clinical phenotypes of sarcoidosis based on the distribution of lesions, with a primary focus on organ involvement. A large cohort study conducted in Spain that included 1230 patients found that those with pulmonary involvement had lower rates of skin and salivary gland disease but a higher frequency of liver involvement [21]. A retrospective multicenter study identified five clinical phenotypes using hierarchical cluster analysis. Their analysis revealed that cardiac and fibrotic pulmonary involvement clustered within the same phenotype, which also included neurological and abdominal manifestations [22].

Considering the superiority of FDG PET in the diagnosis of CS, it is expected that this modality will contribute to phenotypic disease characterization in sarcoidosis [23]. Papiris et al. proposed four distinct phenotypes of systemic sarcoidosis based on organ involvement: (1) thoracic nodal (hilar–mediastinal), (2) thoracic nodal and pulmonary, (3) extended thoracic and extrathoracic nodal (including inguinal, abdominal, and supraclavicular stations), and (4) a systemic phenotype involving all of the above plus organs such as muscle, bone, spleen, and skin [24]. Abdominal LN involvement is characteristic of phenotypes 3 and 4. No significant differences in myocardial involvement were found among the identified clusters, which suggests that CS may occur independently of other organ-based phenotypes.

Despite the comprehensive evaluation performed, this study has several limitations. First, it is retrospective in nature, which may have introduced selection bias and limited control over confounding variables. Second, while FDG-PET/CT is highly sensitive, FDG uptake is not specific to sarcoidosis and can be influenced by infections, malignancies, or other inflammatory processes. Histopathological confirmation was not available for all FDG-avid LNs, which limits correlation between imaging findings and definitive diagnosis. We used decreased or resolved FDG uptake on follow-up PET/CT as supportive evidence to increase confidence that an FDG-avid lesion represented true sarcoidosis. This was not used to diagnose sarcoidosis itself, but rather to strengthen the interpretation of each lesion as a true-positive finding. The heatmap provide valuable visual and quantitative insights but do not account for dynamic disease processes or temporal changes in nodal involvement. In addition, variability in image interpretation and differences in PET scanner technology or protocols could have affected SUV measurements and lesion detectability, even though we confirmed that the recovery coefficients were nearly equivalent across the PET/CT scanners based on a NEMA phantom study. Finally, the cohort was derived from patients undergoing evaluation for suspected CS, which may not fully represent the broader sarcoidosis population. In our country, FDG-PET is primarily approved for the diagnosis of CS, and it may account for the low incidence of lung uptake observed in our study.

## Conclusion

This study conducted a comprehensive assessment of lymph node involvement patterns on FDG-PET/CT in patients with known or suspected CS. Distinct dissemination patterns were identified, highlighting the lymphatic spread of sarcoidosis into the upper abdominal region. These observations contribute to a deeper understanding of the disease’s pathophysiology and hold potential value for improving diagnostic evaluation.
